# Alcohol Hangover and Multitasking: Effects on Mood, Cognitive Performance, Stress Reactivity, and Perceived Effort

**DOI:** 10.3390/jcm9041154

**Published:** 2020-04-17

**Authors:** Sarah Benson, Elizabeth Ayre, Harriet Garrisson, Mark A Wetherell, Joris C Verster, Andrew Scholey

**Affiliations:** 1 Centre for Human Psychopharmacology, Swinburne University, Melbourne VIC 3122, Australia; sarahbenson@swin.edu.au (S.B.); eayre@swin.edu.au (E.A.); mark.wetherell@northumbria.ac.uk (M.A.W.); J.C.Verster@uu.nl (J.C.V.); 2 Stress Research Group, Department of Psychology, Northumbria University, Northumberland Building, Newcastle upon Tyne NE1 8ST, UK; 3 Division of Pharmacology, Utrecht Institute for Pharmaceutical Sciences (UIPS), Utrecht University, 3584CG Utrecht, The Netherlands

**Keywords:** hangover, alcohol, internet, attention, executive function, working memory

## Abstract

The aim of this study was to examine the effects of hangover on mood, multitasking ability, and psychological stress reactivity to cognitive demand. Using a crossover design and semi-naturalistic methodology, 25 participants attended the laboratory in the morning following a night of (i) alcohol abstinence and (ii) alcohol self-administration during a typical night out (with order counterbalanced across participants). They completed a four-module multitasking framework (MTF, a widely used laboratory stressor) and a battery of questionnaires assessing mood, hangover symptom severity, and previous night’s sleep. The effects of the MTF on mood and perceived workload were also assessed. Participants in the hangover condition reported significantly lower alertness and contentment coupled with a higher mental fatigue and anxiety. Multitasking ability was also significantly impaired in the hangover condition. Completion of the cognitive stressor increased reported levels of mental demand, effort, and frustration, and decreased perceived level of performance. MTF completion did not differentially affect mood. Lastly, participants rated their sleep as significantly worse during the night prior to the hangover compared with the control condition. These findings confirm the negative cognitive and mood effects of hangover on mood. They also demonstrate that hangover is associated with greater perceived effort during task performance.

## 1. Introduction

The effects of binge drinking on cognitive functioning beyond intoxication, to the subsequent alcohol hangover, has received increasing attention over the past few years. The alcohol hangover is generally obtained following alcohol intake equivalent to a blood alcohol concentration (BAC) of at least 0.10%–0.12% [1], although it can occur following lower BACs [2], and commences once BAC approaches 0.00% [3]. It is characterized by a cluster of physical symptoms, negative mood, and impaired cognitive functioning. Commonly reported symptoms include headache [1,4], nausea [1,5], anxiety [5,6,7], fatigue [1,6,8], reduced alertness [1,6,9,10], and concentration difficulties [1,11,12].

Hangover-induced cognitive impairment has been described across several domains including spatial and visual abilities [13], attention [14,15,16,17], memory, information processing [18], and reaction time [18,19,20]. Conversely, some studies have failed to find convincing evidence for hangover-induced cognitive impairment [10,11,16]. A recent meta-analysis of 11 articles reported hangover-induced impairment to short and long-term memory, psychomotor performance, and sustained attention, but not divided attention [21]. As suggested by the authors, accumulating mental fatigue caused by prolonged attentional demand is more evident in sustained as opposed to divided attention tasks that may drive the differential attention effects [22].

Importantly, each of the studies [15,17,23,24] included in the meta-analysis [21] assessing the effects of hangover on divided attention used tasks with relatively few stimuli and limited task-switching. Furthermore, three of the four studies were published decades ago and appear under-powered [15,23,24]. More recent studies have utilized assessments of compound behaviors requiring more complex divided attention across several stimuli, such as simulated driving [25,26] and flying [27,28] ability. These have consistently demonstrated hangover-induced performance impairment. Indeed, driving and flying are complex skills which require several cognitive processes and as such, are not strict measures of divided attention per se. Nevertheless, complex tasks are a realistic representation of real-world activities where attention is often divided across several stimuli streams, causing cognitive demand [29] and a stress reactivity response [30], characterized by increased negative mood, cortisol, and self-reported stress [30,31].

Stress reactivity is increased in certain populations such as recreational drug users [32]. Given that the alcohol hangover is characterized by worsened cognition and mood, individuals with a hangover may display an exaggerated response to stress. The effects of hangover on stress reactivity have not been previously assessed but warrant investigation. Particularly in the context of potential negative implications of performing everyday multitasking and potentially stress-inducing activities, such as driving, studying, or working, with a hangover.

Real-life stressors typically involve simultaneous exposure to multiple stressors. The Purple multitasking framework (MTF) requires simultaneous attention and response to several stimuli and, therefore, may have better ecological validity than previously used divided attention tasks [33]. The MTF has previously been shown to elicit a response typical of workload stress (where mental resources cannot meet ongoing demands). That is reduced self-rated calmness, elevated stress, and state anxiety coupled with increased perceived demand, effort, and frustration [32,33,34].

The aim of this investigation was to examine the effects of hangover on mood, stress reactivity, multitasking performance, and perceived effort of performing the tasks. Specifically, we hypothesized that compared with no hangover, hangover would be associated with more negative mood, higher stress reactivity, poorer performance, and greater perceived effort during multitasking.

## 2. Experimental Section

### 2.1. Method

The present study was approved by the Swinburne University Human Research Ethics Committee (SUHREC, approval number 2016/061) and was conducted in accordance with the Declaration of Helsinki.

### 2.2. Design

This study utilized a semi-naturalistic, crossover design whereby, in the hangover condition, participants consumed alcohol during a ‘typical’ night out and attended the laboratory the following morning. The testing visit of the no-hangover condition was held following a night of alcohol abstinence.

### 2.3. Participants

Thirty-six participants enrolled in the study. However, seven participants failed to complete the two testing visits and one participant reported alcohol intake the evening prior to the hangover-free visit and thus, was excluded. The final sample consisted of 25 participants (76% female) with a mean age of 25.32 years (range = 18–35 years).

All participants were healthy, not taking any medications that could potentially interact with alcohol and experienced an alcohol hangover frequently. Exclusion criteria were current or past alcohol abuse and current or past psychiatric disorders.

### 2.4. Measures

#### 2.4.1. Blood Alcohol Concentration (BAC)

To ensure a BAC of 0.00% prior to the commencement of each testing visit, BAC readings were recorded using a regularly calibrated Lion Alcolmeter SD400PA.

#### 2.4.2. Consensus Sleep Diary (CSD)

All sleep parameters were collected using the 9-item CSD [35].

#### 2.4.3. Alcohol Consumption Questions

Participants were questioned on their alcohol intake on the evening prior to the testing visits. Specifically, participants were asked the number of standard drinks of various drink types (i.e., beer, cider, wine, spirits, and alcohol mixed with energy drink) that were consumed and the number of hours that they had spent drinking.

#### 2.4.4. Estimated BAC (eBAC)

Responses to the alcohol consumption questions were used to calculate eBAC. eBAC obtained on the night of drinking was calculated by averaging the total body water (TBW) estimates of Forrest [36], Watson [37], Seidl [38], Widmark [39], and Ulrich [40] (males only) calculations. The mean TBW was then used in the following eBAC formula:

BAC = (G/(TBW) − β × t(1)

where G is the amount of alcohol consumed in grams; β is the metabolic rate in gram per hour; and t is time in hours.

#### 2.4.5. Multitasking Framework

**Figure 1:**
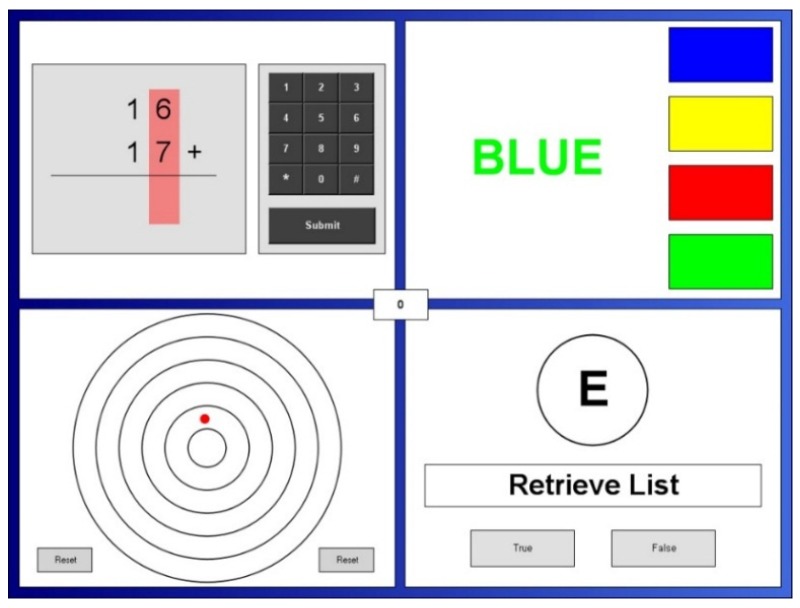
Layout of the Purple multitasking framework (MTF). The task requires participants to simultaneously perform four cognitive tasks. These were (clockwise from top left) Mental Arithmetic, Stroop, Letter Search, and Visual Tracking.

The Purple multitasking framework (Purple MTF: Purple Research Solutions, UK) has been shown to induce cognitive demand, stress, negative mood, and anxiety [32,33,41,42,43]. The task requires attention to be given to four tasks that are presented simultaneously, each on one of four quadrants on a computer screen (see Figure 1).

In the centre of the screen, a counter displaying the overall performance score, based on accuracy and reaction time, is presented. The tasks used in the current study were Mental Arithmetic, Stroop, Letter Search, and Visual Tracking. Each task was set to the ‘difficult’ level and the battery was completed over 20 min (for a detailed description of the tasks and scoring, please see [44]). As well as an overall score, each task was scored separately.

#### 2.4.6. NASA-Task Load Index (NASA-TLX)

Participants were asked to provide their subjective assessment of the workload presented by the Purple MTF using the NASA-TLX [45]. Workload was assessed on six dimensions: Mental Demand, Physical Demand, Temporal Demand, Own Performance, Effort, and Frustration.

#### 2.4.7. Single Item Hangover Symptom Severity Score

Overall hangover severity was measured using a single visual analogue scale (VAS) asking participants to rate ‘how severe is your hangover’ on a scale from 0, being ‘no hangover symptoms, to 10, being ‘very severe hangover symptoms’.

#### 2.4.8. Alcohol Hangover Severity Scale (AHSS)

The AHSS [46] measures hangover severity according to 12-items measured on a 10-point Likert scale. The AHSS is found to be reliable (Cronbach’s α = 0.85) and valid (*r* = 0.92 correlation with the Acute Hangover Scale (AHS; [4])) [46].

#### 2.4.9. Stress and Fatigue Visual Analogue Mood Scale (Stress and Fatigue VAMS)

Self-reported levels of stress and fatigue were each measured using a single-item visual analogue scale labeled stress and fatigue with the endpoints labeled ‘not at all’ and ‘extremely’.

#### 2.4.10. Spielberger State-Trait Anxiety Inventory, State Portion (STAI-S)

The STAI consists of two parts evaluating trait and state anxiety, respectively. This study used the state (STAI-S; [47]) portion only. Participants responded to 20 statements (e.g., “I feel calm”, “I am relaxed”) on a 4-point scale ranging from ‘not at all’ to ‘very much so’. Scores are combined to give a measure of current anxiety.

#### 2.4.11. Bond-Lader Visual Analogue Mood Scales (Bond-Lader VAMS)

The Bond-Lader VAMS [48] have been utilized in numerous pharmacological, psychopharmacological, and medical trials. These scales comprise a total of 16 lines measuring 100 mm long and anchored at either end by antonyms (e.g., alert-drowsy, calm-excited). Participants indicate their current subjective position between the antonyms on the line. Individual item scores were calculated as the distance along the line. Outcomes are three factor analysis-derived scores: Alertness, calmness, and contentment.

### 2.5. Procedure

Participants were required to attend a screening visit and two testing visits; one while experiencing a hangover and one following at least 24-h of alcohol abstinence. During the screening visit, participants provided a written informed consent before being assessed for eligibility. Participants then underwent training and practice in completing the Bond-Lader VAMS and Stress and Fatigue VAMS, a cognitive dual attention task (to be reported elsewhere), the Purple MTF, and the NASA-TLX.

Participants were instructed not to consume any caffeine the morning of their testing visits or any alcohol within 24-h prior to the no-hangover visits. Participants were also asked for the food consumed prior to the testing visits to be held consistent. Lastly, participants booked in their first testing visit, which could be either the hangover or no-hangover condition, depending on the participants planned drinking activities.

At the commencement of each testing visit, participants were breathalyzed to ensure a BAC of 0.00%. Participants then completed the pre-stressor Bond-Lader VAMS, the individual Stress and Fatigue VAMS, and the STAI-S. They then underwent the Purple MTF, followed by the post-stressor Bond-Lader VAMS, Stress and Fatigue VAMS, and STAI-S. Finally, they completed the NASA-TLX, alcohol consumption questions, the single-item measure of overall hangover severity, the AHSS, and lastly, the CSD.

### 2.6. Statistical Analysis

All analyses were performed using SPSS version 25 (IBM Corp, Armonk, NY, USA). The effects of the Purple MTF on the three mood dimensions from the Bond-Lader VAMS (‘alert’, ‘calm’, ‘content), anxiety measured using the STAI-S, and the individual VAMS items (‘stress’, ‘mental fatigue’) were assessed using two-way (Hangover; present, absent × Time; pre, post) repeated measures ANOVA. All other variables were analyzed using paired sample *t*-tests comparing hangover with control conditions.

Effect sizes (Cohen’s d) were calculated for all significant *t*-test findings using ‘Equation 8’ [49] to account for dependence in the data. All testing was two-tailed, and comparisons were planned prior to testing.

## 3. Results

### 3.1. Consensus Sleep Diary (CSD)

In the hangover condition, participants rated their sleep quality as significantly worse (*t*(24) = 2.70, *p* = 0.012, *d* = 0.54) and reported significantly more awakenings (*t*(24) = 2.47, *p* = 0.021, *d* = 1.80) than in the control condition. However, the length of awakenings and time taken to fall asleep did not differ between the conditions.

### 3.2. eBAC

The mean eBAC level on the night prior to the hangover visit was 0.135% (±0.001%).

### 3.3. Hangover Symptom Severity

As shown in Table 1, at the hangover visit, participants were significantly negatively affected on each hangover symptom included in the AHSS apart from ‘sweating’.

### 3.4. Multitasking Performance

The total MTF score was significantly lower in the hangover compared with the no-hangover condition (*t*(24) = 2.26, *p* = 0.033, *d* = 0.46). Performance on the four individual tasks did not differ with condition.

### 3.5. Mood

**Figure 2:**
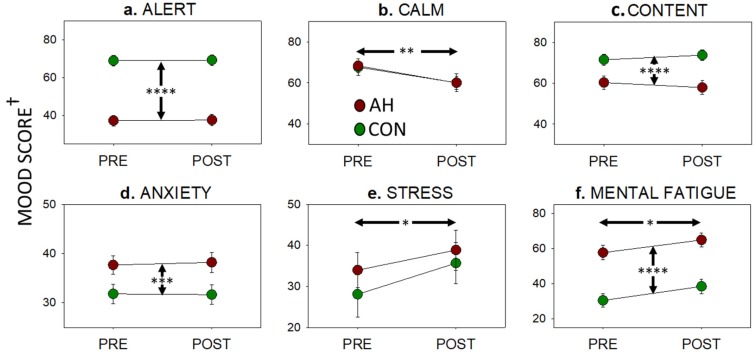
Effects of hangover and completing the Purple MTF on dimensions of mood measured pre- and post-Purple MTF. (**a**) Alert, (**b**) calm, and (**c**) content are derived from the Bond-Lader scales; (**d**) anxiety is derived from the Spielberger State-Trait Anxiety Inventory (STAI state portion); (**e**) stress and (**f**) mental fatigue are single visual analogue scales. Graphs depict means with standard errors of the mean (SEM). Vertical and horizontal arrows indicate a significant main effect of hangover and time, respectively (*, *p* < 0.05; **, *p* < 0.01; ***, *p* < 0.005; ***, *p* < 0.001). ^†^ Scales range from 0–100 except (**d**) anxiety which ranges from 20–80.

As displayed in Figure 2, completion of the Purple MTF did not differentially affect mood ratings in either hangover condition. However, there were significant main effects of hangover on alertness (F(1,24) = 54.63, *p* < 0.001), contentedness (F(1,24) = 16.34, *p* < 0.001), anxiety (F(1,24) = 10.97, *p* = 0.003), and mental fatigue (F(1,24) = 40.70, *p* < 0.001). There were also significant main effects of time (pre-post) on calmness (F(1,24) = 8.42, *p* = 0.008, stress (F(1,24) = 5.86, *p* = 0.023), and mental fatigue (F(1,24) = 5.90, *p* = 0.023). Prior to completing the Purple MTF, participants reported significantly worse levels of alertness (*t*(24) = 7.33, *p* <.001, *d* = 1.47), contentedness (*t*(24) = 3.34, *p* = 0.002, *d* = 0.68), mental fatigue (*t*(24) = 4.90, *p* < 0.001, *d* = 0.99), and anxiety (*t*(24) = 2.58, *p* = 0.016, *d* = 0.21) in the hangover, compared to the no-hangover condition.

### 3.6. NASA-TLX

**Figure 3:**
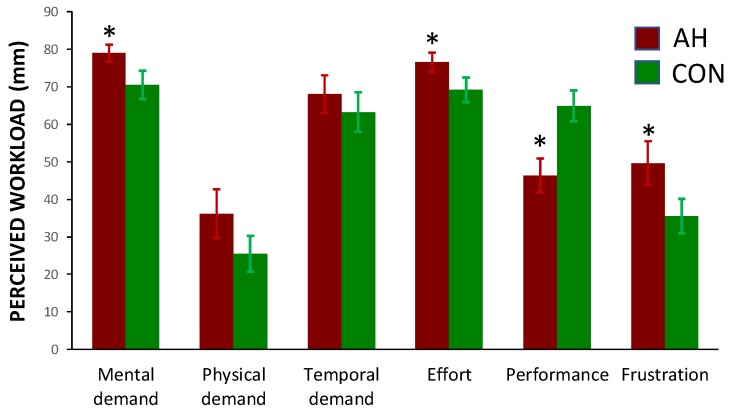
Mean (standard error) perceived levels of workload demand in the hangover (AH) and control (CON) conditions. * *p* < 0.05 between conditions.

Participants rated the workload demand of completing the Purple MTF as more mentally demanding (*t*(24) = 2.19, *p* = 0.039, *d* = 0.46) and effortful (*t*(24) = 2.29, *p* = 0.031, *d* = 0.47) during the hangover condition. Additionally, participants rated their performance as worse (*t*(24) = 2.75, *p* = 0.011, *d* = 0.55) and the task as more frustrating (*t*(24) = 2.26, *p* = 0.033, *d* = 0.46) in the hangover condition, see Figure 3.

## 4. Discussion

The current study assessed the effects of alcohol hangover on mood, multitasking performance and stress reactivity, and perceived demand. Several of our hypotheses were supported. Compared with no hangover, hangover was associated with significantly more negative mood (lower alertness and contentment, higher anxiety, and mental fatigue). Additionally, hangover was associated with poorer multitasking performance and greater perceived effort during multitasking. Counter to our hypothesis, stress reactivity was not differentially affected by hangover.

Hangover was reliably induced by using a semi-naturalistic study design and allowing participants to self-administer alcohol, according to the estimated BAC calculations, participants obtained a mean eBAC of 0.135%, a level beyond that deemed required to cause a hangover [1,50]. Self-reports also provided support for the link between hangover and poor sleep quality [16,51,52]. However, our findings were inconsistent with previous sleep studies that show a faster sleep onset after alcohol [53,54,55].

The negative mood associated with hangover was manifested as significantly lower alertness and contentment, and higher anxiety and mental fatigue. These findings are consistent with symptoms commonly reported in the hangover literature [1,5,6,7,8,9,10]. They are also supported by significant effects, here and elsewhere, on individual items on the AHSS which gauge elements of mood—namely fatigue, apathy, concentration problems, and confusion.

Participant self-reports also confirmed the presence of a hangover and hangover symptoms, demonstrating that the manipulation was successful. Every item of the AHSS differed significantly between visits with the exception of ‘sweating’ (*p*-value for ‘sweating’ = 0.055 two-tailed). Inspection of the effect sizes shows that the largest effect by far is for the single hangover item (Cohen’s *d* = 3.07). This adds further support to the recent argument that the single item measure can capture hangover better than more granular items [56].

The second most prominent effect (*d* = 2.20) was for ‘concentration problems’. This supports the documented cognitive impairment seen with hangover. In the current study, this was manifested as significantly poorer overall performance on the MTF but not on any individual task thereof. These results, along with previous evidence for hangover-induced impairment to more complex [25,26,27,28] but not simple divided attention [15,17,23,24] suggest that hangover differentially impairs tasks which draw on multiple domains. This result may help explain certain inconsistencies in the literature on hangover and cognitive performance (as outlined in the introduction). Unlike previous studies that have utilized assessments of divided attention across relatively few stimuli with limited task-switching, the current study used a cognitive stressor with multitasking across four tasks. Unlike many other laboratory stressors, this model has better correspondence with the more usual day-to-day stress that typically requires attention and response to several tasks concurrently [57]. Additionally, it may be that behavioral outcomes which are aggregates of several cognitive domains may be most sensitive to the negative effects of hangover. This has clear implications for behaviors such as driving which draw on several cognitive domains simultaneously. Further research is needed utilizing cognitive measures which mirror the processes involved in driving.

There are additional layers of cognitive control and function that are required for complex multitasking. For example, multitasking places a considerable load on working memory and executive processes, in addition to the resources needed for task performance, thus, likely taxing cognitive capacities [58,59]. These data suggest that hangover depletes cognitive resources making the effects of cognitive demand more profound than in a no hangover state.

Contrary to our hypothesis, completion of the cognitive stressor failed to elicit further changes in mood. The fact that two of the mood measures (calmness and stress) were significantly worse due to completing the MTF (independent of hangover condition) strongly suggests that the measure is sensitive to change in this cohort. Inspection of the pattern of results suggests that, for the other measures, the mood state of participants prior to completion of the MTF was already low and unlikely to change further.

Supporting our hypothesis, NASA-TLX scores revealed that being exposed to the cognitive stressor resulted in greater subjective demand in the hangover group. Compared with the control condition, hangover significantly increased ratings of perceived effort, mental demand, and frustration. Additionally, participants were aware of their poorer performance in the hangover condition. This is unlike alcohol intoxication, where individuals become ‘uncalibrated’ and, thus, overestimate their performance [60]. Despite awareness of poorer performance, it appears that in the hangover state individuals are unable to draw on additional cognitive resources to compensate and meet ongoing task demands. This has clear implications for behaviors requiring complex multitasking—including driving.

The physiological mechanisms of hangover are not well understood, but likely involve multiple processes [61], including actual and perceived immune status [62,63].

Limitations of this study included relying on self-reported alcohol intake and eBAC calculations to estimate BAC obtained to induce the hangover, and the lack of objective stress (i.e., cortisol) and sleep (i.e., actigraphy) measurement. Since excessive drinking commonly results in memory impairment [64,65,66], participants’ accuracy of recalling their drinking behaviors may be questioned, a limitation inherent in hangover semi-naturalistic and naturalistic study designs (see [2]). Given the lack of empirical investigation into the effects of hangover on “real-life” multitasking ability and stress reactivity, this study fills an important research gap.

## 5. Conclusions

The findings presented here show that hangover negatively affects performance on an ecologically valid, complex multitasking tasks and mood. This is the first report of the effects of hangover on psychological stress reactivity and results indicated that, despite a greater perceived workload in the hangover condition, mood, and/or stress were not differentially affected following the completion of the cognitively demanding task. Future research should include a variety of complex ‘real-life’ measures of cognition and be directed towards determining the physiological changes that occur with a hangover.

## Figures and Tables

**Table 1:** Effects of alcohol hangover on the single item severity score and items from the Alcohol Hangover Severity Scale (AHSS). Means and standard deviations are presented with *t*-statistic, associated *p*-value, and Cohen’s d effect size.

Item	Condition		*t*-Value	*p*-Value	Cohen’s d
	Control	Hangover			
**Single-Item Severity Scale**					
‘How severe is your hangover?’	0.05(0.16)	4.67(2.36)	10.07	<0.001	3.07
**Alcohol Hangover Severity Scale**					
Fatigue	2.40(2.36)	7.36(2.18)	9.08	<0.001	1.82
Apathy	0.56(0.96)	3.64(2.80)	5.69	<0.001	1.35
Concentration problems	1.00(1.41)	5.88(2.22)	10.54	<0.001	2.20
Clumsiness	0.68(0.90)	4.72(3.14)	6.46	<0.001	1.54
Confusion	0.48(1.12)	3.48(3.20)	5.30	<0.001	1.38
Thirst	1.44(2.20)	6.24(2.51)	8.86	<0.001	1.78
Sweating	0.40(1.04)	1.20(1.94)	2.02	0.055	-
Shivering	0.16(0.62)	1.84(3.00)	2.80	0.010	0.69
Stomach pain	0.08(0.28)	2.36(2.84)	4.04	<0.001	1.10
Nausea	0.40(1.00)	3.12(2.76)	4.95	<0.001	1.14
Dizziness	0.36(0.91)	4.28(2.64)	7.50	<0.001	1.75
Heart pounding	0.40(1.23)	2.24(2.52)	3.71	0.001	0.82
Total score	0.70(0.74)	3.86(1.83)	8.90	<0.001	2.03
